# Parasite contamination of soil in different Peruvian locations and outside built environments

**DOI:** 10.1186/s13071-025-06762-7

**Published:** 2025-04-05

**Authors:** Carlos Pineda, Maritza Dalí Camones Rivera, Eddyson Montalva Sabino, Lucia Estela Mejia, Katherine Elizabeth Keegan, Lizbet Pilar Patricio Alvarez, Javier Jorge Mora, Fernanda Espinoza Vega, Emilio Rey Mejia, Patrick Olivas Herrera, Elisa Palomino Pando, Zhen Zeng, Athos Silva De Oliveira, Maria Jose Villar Mondragon, Barton Slatko, Eric J. Wetzel, Rojelio Mejia

**Affiliations:** 1 Facultad de Medicina Veterinaria y Zootecnia, Universidad Nacional Hermilio Valdizán, Huánuco, Peru; 2 Instituto de Investigación en Enfermedades Tropicales, Universidad Nacional Toribio Rodríguez de Mendoza de Amazonas, Chachapoyas, Peru; 3 Laboratory of Human and Environmental Parasitology, Pediatrics Tropical Medicine, Baylor College of Medicine, Houston, TX USA; 4 Department of Pharmaceutical Health Outcomes and Policy, College of Pharmacy, University of Houston, Houston, TX USA; 5 Department of Biology and Global Health Initiative, Wabash College, Crawfordsville, IN USA; 6 Robert Turner College and Career High School, Pearland, IX USA

**Keywords:** Parasitic DNA, Soil, qPCR

## Abstract

**Background:**

Soil is a reservoir for many parasites that can affect human and animal health, especially in tropical regions where soil-transmitted helminths and protozoa thrive. Understanding how environmental factors influence parasite distribution will provide a basis for relating how climate changes may intensify their impacts, altering parasite habitats and increasing transmission risks. We surveyed soil parasite prevalence, burden, and diversity in several different Peruvian environmental ecologies to catalog current parasite presence and provide a baseline for future surveys.

**Methods:**

A total of 198 soil samples from 43 locations across three Peruvian regions—Tingo María (TM) (Amazon rainforest), Andabamba/Marabamba (A/M) (Andean highlands), and Huánuco city parks—were analyzed using multi-parallel quantitative real-time polymerase chain reaction (qPCR) to detect soil-transmitted helminths (STH) and protozoan DNA from entry, patio, and latrine sites.

**Results:**

Parasites were detected in 93% of locations, with 84% showing polyparasitism. TM houses had a higher odds ratio of contamination with *Ascaris lumbricoides* and *Trichuris trichiura* than those in A/M. TM also showed significantly higher odds of helminth contamination in patios than entries. TM had significantly more parasite species, with helminth species significantly higher in the patio versus entry. A/M had higher protozoan prevalence with *Blastocystis* species, with a greater odd ratios to TM. A/M had an increase of *Acanthamoeba* species in patios versus entries, indicating a niche favoring protozoans in these arid conditions.

**Conclusions:**

The observed variability in soil parasite prevalence between tropical rainforest and highland regions highlights the influence of environmental niches on parasite distribution, which may shift further due to climate change. This study demonstrates a sensitive approach to monitoring environmental contamination with parasites by leveraging qPCR. The findings underscore the importance of ecological surveillance for assessing parasitic transmission risks, which is crucial for guiding public health interventions, especially as environmental changes accelerate.

**Graphical abstract:**

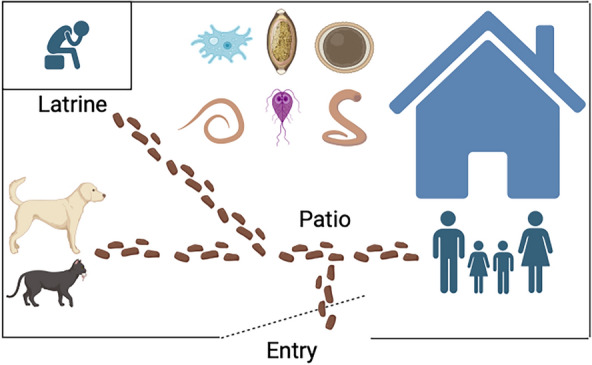

**Supplementary Information:**

The online version contains supplementary material available at 10.1186/s13071-025-06762-7.

## Background

Soil-transmitted helminths (STH) and protozoan parasites can lead to significant health problems in tropical and subtropical areas worldwide, including malnutrition, anemia, and growth delays in children [1]. These parasitic infections are caused by helminths (*Ancylostoma* species, *Ascaris lumbricoides*, *Necator americanus*, *Strongyloides stercoralis*, *Trichuris trichiura*, *Toxocara cati*, *Toxocara canis*, *Taenia solium*) and protozoa (*Blastocystis* species, *Entamoeba histolytica*, *Giardia intestinalis*, and *Cryptosporidium* species). Other soil-borne organisms, such as *Acanthamoeba* species, can be opportunistic human pathogens, the most environmentally prevalent free-living protozoa [2]. In terms of STH, upwards of 20% of the world’s population (1.5 billion people) are infected, leading to an estimated 5.2 million disability-adjusted life years (DALYs) [3].

Risk assessments can be performed based on parasite soil contamination from environmental sites using a standardized multi-parallel real-time quantitative polymerase chain reaction (qPCR) method for detecting parasite DNA [4–6]. We aimed to detect STH and protozoan parasite DNA in soil collected from two environmentally distinct areas in Peru: Tingo María, in the Selva Alta (“High Forest”) of Peru’s Eastern Piedmont (600 m elevation), and Andabamba/Marabamba (mountainous Andean highlands, 1900 m elevation) and Huánuco (mountainous basin Andean highlands, 1900 m elevation). The area of Tingo María is the gateway to the biodiverse Tingo María National Park, and the city of Tingo María lies in an intermediate geographical zone known as the ceja de selva (“eyebrow of the jungle”) (Additional file 1: Fig. S1). This region has a tropical rainforest climate, typically hot, humid, and wet with no dry season. In contrast, the Andabamba/Marabamba area, including nearby Huánuco, has a semi-arid climate, comprising parts of the Andean highlands and the High Jungle (mountain rim) regions (Additional file 1: Fig. S1). It has mild weather with an average annual temperature of 20 °C (68 °F). These sites were selected primarily because of their different soil environments to ascertain similarities and differences between helminth and protozoan soil prevalence and burden. Local subsistence farming and decentralized waste disposal could contribute to soil parasite contamination. The study sites were all located in rural resource-limited areas. Climate change leading to soil environmental shifts will likely change these parasitic parameters, leading to consequences for parasite risk monitoring and subsequent health care approaches [7, 8].

## Methods

### Study locations

This cross-sectional study included 198 samples taken from 43 independent locations as part of an ongoing public health initiative by the Universidad Nacional Hermilio Valdizan, Peru. These three locations were chosen to represent different environmental ecologies, as described above. At each location, two to seven soil samples were collected (76 from 14 houses in Tingo María, 77 from 22 houses in Andabamba/Marabamba, and 45 from seven parks in Huánuco) from outdoor built environments, including entries, patios, and outdoor latrines (Additional file 2: Fig. S2) in the cases of Tingo María and Andabamba/Marabamba. Households were randomly selected after obtaining consent from the owners. Latrines were limited-service latrines on the Joint Monitoring Programme (JMP) sanitation ladder about 2 m from the house’s back door [9]. The sampling size provided a baseline for parasite identity and diversity.

### DNA extraction from soil

Each sample was processed at the Universidad Nacional Hermilio Valdizán (UNHEVAL), Huánuco, Peru, using strict protocols to eliminate cross-contamination. To minimize the risk of cross-contamination during DNA extraction and qPCR, all reagents and samples were prepared in a CleanPrep workstation (Mystaire, Creedmoor, NC, USA). Up to 50 g of wet soil was collected and stored at −20 °C until use. Each sample was weighed and resuspended to 45 ml in phosphate-buffered saline (PBS; Alfa Aesar, Ward Hill, MA, USA) containing 0.05% Tween (Sigma-Aldrich, St. Louis, MO, USA) in a conical centrifuge tube. The samples were then shaken and inverted for 5 min and centrifuged at 500×*g* for 5 min. Supernatants were then decanted and discarded. A total of 10 ml of sugar solution (320 g granulated sugar in 620 ml distilled water, specific gravity of 1.30) was added to the pellets for parasite egg/larvae/cyst flotation. After shaking and inversion for 5 min, samples were centrifuged at 500×*g* for 5 min. Supernatants were then filtered through a mixed cellulose ester (MCE) membrane (3.0 µM, MF-Millipore, Merck KGaA, Darmstadt, Germany), and the resultant filtrates were processed using the FastDNA SPIN Kit for Soil (MP Biomedicals, Santa Ana, CA, USA) as described previously [4–6]. An external DNA control, unrelated to the target parasites, was added to each sample before the purification step to allow for the quantification of the isolated DNA using PCR [10]. The eluent was spotted on 0.2 µm filter paper (Millipore, Merck KGaA, Darmstadt, Germany) and air-dried before being shipped at ambient temperature to Baylor College of Medicine, Houston, TX, USA. Once received, DNA was extracted from the filter papers by overnight room-temperature elution using 100 µl of elution buffer (MP Biomedicals).

### Multi-parallel quantitative real-time PCR

Recovered DNA was analyzed by a multi-parallel qPCR as described previously [4–6], with the addition of a *T. solium* (TsolR13) primer and probes [11] (Additional file 4: Table S2). Samples were run on a QS7 Pro Fast Real-time PCR System (Applied Biosystems, Waltham, MA, USA), and plasmid samples containing the target sequences were serially diluted to create the standard curve (duplicates) [12]. Only a cycle threshold (Ct) of 38 or lower was considered positive for all parasites based on spiking studies using parasite egg/larvae/cyst and detection using qPCR (Additional file 3: Table S1) [4, 5].

Parasite concentrations of DNA (fg/µl) were calculated by linear regression, including those outside the dynamic range of the standard curve (Design and Analysis Software 2.6.0, Thermo Fisher Scientific, USA). The exogenous internal DNA control was used to assess the quality of DNA extraction and inhibition efficiency in all samples [10]. Singleplex qPCR was used on all samples to detect only one parasite in each 96-well plate. Each sample plate was validated with a positive (parasite DNA) and a negative (no DNA) control. A built environment was considered contaminated with a parasite if at least one sample from a given environmental built location/house or park was qPCR-positive.

Statistical methods used for this study include the Mann–Whitney *t*-test and the Kruskal–Wallis analysis of variance (ANOVA) test with Dunn’s correction to compare locations and parasite concentrations. Odds ratios were calculated by logistic regression, Fisher exact, or Firth’s bias-reduced logistic regression when the sample size of one cell was less than five. Values were considered significant if *P* < 0.05.

## Results

### Prevalence among all three sample areas

Of the three sample areas, 93.0% (40/43) of houses and parks tested positive for at least one parasite, with 83.7% (36/43) showing polyparasitism. Up to seven different parasites were detected in an individual house or park site (range 0–7). For STH, 53.5% (23/43) of houses and parks were positive, with 14% of the sites (6/43) positive for *Ancylostoma* species, 16.3% (7/43) for *A. lumbricoides*, 7% (3/43) for *N. americanus*, 16.3% (7/43) for *S. stercoralis*, 2.3% (1/43) for *T. solium*, 4.7% (2/43) for *T. canis*, and 16.3% (7/43) for *T. trichiura*. No *T. cati*-positive sample was found. For protozoa, 88.4% (38/43) of houses and parks were contaminated. Among these, 58.1% (25/43) were positive for *Acanthamoeba* species, 81.4% (35/43) were positive for *Blastocystis* species, and 18.6% (8/43) were positive for *G. intestinalis*. No *Cryptosporidium* species or *E. histolytica* detection was observed (Table 1).

**Table 1:** Prevalence of parasites and DNA concentrations in the soil

Parasite/location	Contamination rate by samples	Contamination rates by sites	DNA concentration in kg of soil (fg/µl), mean (range)
HELMINTHS			
*Ancylostoma* species			
Overall	3.5% (7/198)	14.0% (6/43)	7.61 (0.249–36.7)
Tingo María	2.6% (2/76)	14.3% (2/14)	18.5 (0.249–36.7)
Andabamba/Marabamba	6.5% (5/77)	18.2% (4/22)	3.26 (0.442–12.5)
Huánuco	0% (0/45)	0% (0/7)	0
*Ascaris lumbricoides*			
Overall	6.6% (13/198)	16.3% 7/43)	18.58 (0.156–152.7)
Tingo María	15.8% (12/76)	42.9% (6/14)	20.12 (0.179–152.7)
Andabamba/Marabamba	1.3% (1/77)	4.5% (1/22)	0.116
Huánuco	0% (0/45)	0% (0/7)	0
*Necator americanus*			
Overall	1.5% (3/198)	7.0% (3/43)	0.125 (0.0634–0.227)
Tingo María	0% (0/76)	0% (0/14)	0
Andabamba/Marabamba	2.6% (2/77)	9.1% (2/22)	0.156 (0.0858–0.227)
Huánuco	2.2% (1/45)	14.3% (1/7)	0.0634
*Strongyloides stercoralis*			
Overall	5.6% (11/198)	16.3% (7/43)	0.736 (0.0164–3.52)
Tingo María	14.5% (11/76)	50% (7/14)	0.736 (0.0164–3.52)
Andabamba/Marabamba	0% (0/77)	0% (0/22)	0
Huánuco	0% (0/45)	0% (0/7)	0
*Taenia solium*			
Overall	0.5% (1/198)	2.3%. (1/43)	235.6
Tingo María	0% (0/76)	0% (0/14)	0
Andabamba/Marabamba	0% (0/77)	0% (0/22)	0
Huánuco	2.2% (1/45)	14.3% (1/7)	235.6
*Toxocara canis*			
Overall	1% (2/198)	4.7% (2/43)	133.8 (0.3841–267.2)
Tingo María	1.3% (1/76)	7.1% (1/14)	267.2
Andabamba/Marabamba	1.3% (1/77)	4.5% (1/22)	0.3841
Huánuco	0% (0/45)	0% (0/7)	0
*Trichuris trichiura*			
Overall	3.5% (7/198)	16.3% (7/43)	21.25 (0.01627–104.1)
Tingo María	5.3% (4/76)	28.6% (4/14)	11.16 (0.6382–41.58)
Andabamba/Marabamba	1.3% (1/77)	4.5% (1/22)	104.1
Huánuco	4.4% (2/45)	28.6% (2/7)	0.01921 (0.01627–0.0222)
PROTOZOA			
*Acanthamoeba* species			
Overall	23.7% (47/198)	58.1% (25/43)	0.2037 (0.00688–4.721)
Tingo María	18.4% (14/76)	50% (7/14)	11.16 (0.0098–4.721)
Andabamba/Marabamba	41.6% (32/77)	77.3% (17/22)	0.1343 (0.00688–1.523)
Huánuco	2.2% (1/45)	14.3% (1/7)	0.03401
*Blastocystis* species			
Overall	38.4% (76/198)	81.4% (35/43)	19.99 (0.01424–719.0)
Tingo María	28.9% (22/76)	71.4% (10/14)	49.02 (5.705–719.0)
Andabamba/Marabamba	64.9% (50/77)	95.6% (21/22)	7.175 (0.0558–91.34)
Huánuco	8.9% (4/45)	57.1% (4/7)	20.41 (0.01424–81.52)
*Giardia intestinalis*			
Overall	6.1% (12/198)	18.6% (8/43)	12.87 (1.276–50.92)
Tingo María	5.3 (4/76)	21.4% (3/14)	3.985 (2.589–5.510)
Andabamba/Marabamba	9.1% (7/77)	18.2% (4/22)	19.26 (1.276–50.92)
Huánuco	2.2% (1/45)	14.3% (1/7)	3.628

Considering the 198 samples taken in the 43 independent locations, 58.6% (116/198) tested positive for the presence of DNA from at least one parasite, 24.7% (49/198) showed polyparasitism, and 41.4% (82/198) were negative for any tested parasite. Up to six different parasites were detected in a single sample (range 0–6). Among STH, 19.2% (38/198) tested positive, with 3.5% (7/198) positive for *Ancylostoma* species, 6.6% (13/198) for *A. lumbricoides*, 1.5% (3/198) for *N. americanus*, 5.6% (11/198) for *S. stercoralis*, 0.5% (1/198) for *T. solium*, 1% (2/198) for *T. canis*, and 3.5% (7/198) for *T. trichiura*. No *T. cati* was observed. Among protozoa, 52.5% (104/198) of samples were positive, 23.7% (47/198) for *Acanthamoeba* species, 38.4% (76/198) for *Blastocystis* species, and 6.1% (12/198) for *G. intestinalis*. No *Cryptosporidium* species or *E. histolytica* were detected (Table 1).

### Parasite prevalence in Tingo María

Of the 14 houses sampled in Tingo Maria, 92.9% (13/14) were positive for at least one parasite; 85.7% (12/14) were polyparasitic, with up to seven parasites observed in a single house (range 0–7). Regarding STH, 85.7% (12/14) of houses were positive for at least one species. Specifically, 14.3% (2/14) were positive for *Ancylostoma* species, 42.9% (6/14) for *A. lumbricoides*, 50% (7/14) for *S. stercoralis*, 7.1% (1/14) for *T. canis*, and 28.6% (4/14) for *T. trichiura*. For protozoans, 78.6% (11/14) houses tested positive, with 50% (7/14) for *Acanthamoeba* species, 71.4% (10/14) for *Blastocystis* species, and 21.4% (3/14) for *G. intestinalis* (Table 1) (Additional file 5: Fig. S3).

Regarding the 76 individual samples, 58% (44/76) were positive for the DNA presence of at least one parasite, and 25% (19/76) had more than one parasite detected. Up to six different parasites were detected in a single sample (range 0–6). A total of 32.9% (25/76) were positive for STH: 2.6% (2/76) for *Ancylostoma* species, 15.8% (12/76) for *A. lumbricoides*, 14.5% (11/76) for *S. stercoralis*, 1.3% (1/76) for *T. canis*, and 5.3% (4/76) for *T. trichiura*. Among protozoans, 47.4% (36/76) of samples were positive: 18.4% (14/76) for *Acanthamoeba* species, 28.9% (22/76) for *Blastocystis* species, and 5.3% (4/76) for *G. intestinalis.* No *N. americanus*, *T. solium*, *T. canis*, *T. cati*, *Cryptosporidium* species, or *E. histolytica* was detected (Table 1).

### Parasite prevalence in Andabamba/Marabamba

Regarding the 22 houses sampled in A/M, 100% (22/22) were positive for at least one parasite; 95.5% (21/22) were polyparasitic. Up to four different parasites were detected in a single house (range 1–4) and up to three in a single sample (range 0–3). Among STH, 36.4% (8/22) of houses were positive, with 18.2% (4/22) positive for *Ancylostoma* species, 4.5% (1/22) for *A. lumbricoides*, 9.1% (2/22) for *N. americanus*, 4.5% (1/22) for *T. canis*, and 4.5% (1/22) for *T. trichiura*. Among protozoa, 100% (22/22) of the houses were positive, with 77.3% (17/22) positive for *Acanthamoeba* species, 95.6% (21/22) for *Blastocystis* species, and 18.2% (4/22) for *G. intestinalis* (Table 1) (Additional file 5: Fig. S3).

For the 77 samples from 22 houses, 83.1% (64/77) were positive for at least one parasite, and 36.4% (28/77) had more than one parasite detected. No parasites were detected in 16.8% (13/77) of the samples. Among the 77 samples in A/M, 11.7% (9/77) were positive for STH, and 80.5% (62/77) were positive for protozoa. Among the STH, 6.5% (5/77) were positive for *Ancylostoma* species, 1.3% (1/77) for *A. lumbricoides*, 2.6% (2/77) for *N. americanus*, 1.3% (1/77) for *T. canis*, and 1.3% (1/77) *T. trichiura*. In terms of protozoa, 41.6% (32/77) of samples were positive for *Acanthamoeba* species, 64.9% (50/77) for *Blastocystis* species, and 9.1% (7/77) for *G. intestinalis*. No *Cryptosporidium* species, *E. histolytica*, *T. solium*, *T. cati*, or *S. stercoralis* were detected (Table 1).

### Parasite prevalence in Huánuco

A total of 45 samples were taken from seven city parks in Huánuco. A total of 71.4% (5/7) of parks were positive for at least one parasite, 42.9% (3/7) showed polyparasitism, and up to three different parasites were detected in a single house (range 0–3). Regarding STH, 42.9% (3/7) of parks were positive, with 14.3% (1/7) positive for *N. americanus*, 14.3% (1/7) parks) positive for *T. solium*, and 28.6% (2/7) positive for *T. trichiura*. Among protozoans, 71.4% (5/7) of parks were positive. For *Acanthamoeba* species, 14.3% (1/7) of the parks were positive. For *Blastocystis* species, 57.1% (4/7) of parks were positive. For *G. intestinalis*, 14.3% (1/7) of parks were positive. No positive samples were observed for *Ancylostoma* species, *A. lumbricoides*, *S. stercoralis*, *T. canis*, *T. cati*, *Cryptosporidium* species, or *E. histolytica* (Table 1) (Additional file 5: Fig. S3).

A total of 17.8% (8/45) of the samples were positive for at least one parasite. Regarding STH, 8.9% (4/45) of the samples were positive, with 2.2% (1/45) positive for *N. americanus*, 2.2% (1/45) positive for *T. solium*, and 4.4% (2/45) positive for *T. trichiura*. Among protozoans, 13.3% (6/45) were positive, with 2.2% (1/45) of samples positive for *Acanthamoeba* species, 8.9% (4/45) positive for *Blastocystis* species, and 2.2% (1/45) positive for *G. intestinalis* (Table 1).

### Comparison of parasite burden between sites

**Fig. 1:**
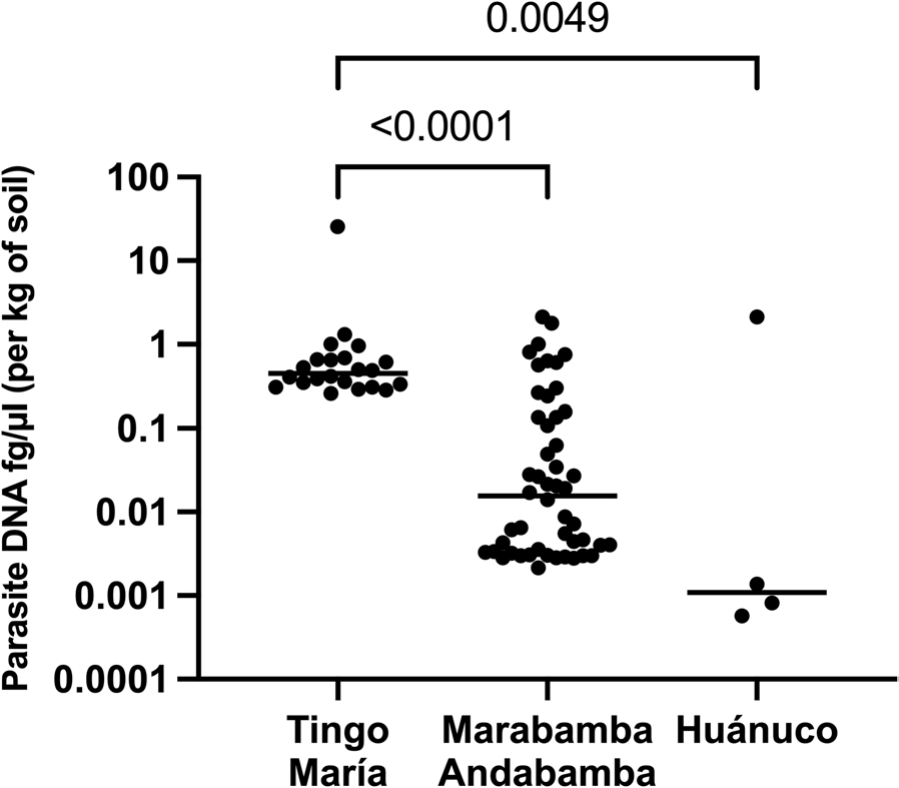
The concentration of *Blastocystis* DNA was significantly higher in Tingo María than in Andabamba/Marabamba, and there was a difference between all three sites (*P* < 0.0001)

Table 1 also presents the mean (range) parasite abundance regarding DNA concentration in kilograms of soil (fg/µl) across the three sites. In Tingo María, the highest DNA concentration of STH parasites was observed for *Ancylostoma* species and *A. lumbricoides*, and among protozoans, *Blastocystis* species had higher concentrations in Tingo María than Andabamba/Marabamba and Huánuco (14.7 vs. 0.4312 vs. 0.2159 (fg/µl)/kg soil, respectively, Kruskal–Wallis *H*-test, *H* = 25.00, *df* = *3*, Dunn’s correction TM vs. MA, *P* < 0.0001, TM vs. Huánuco, *P* = 0.0049) (Fig. 1). In Andabamba/Marabamba, the highest STH DNA concentrations were observed for *T. trichiura*, with *G. intestinalis* being the highest among protozoans. Differences in presence and abundance were observed between Tingo María and Andabamba/Marabamba for *Ancylostoma* species, *T. canis*, *T. trichiura*, and all tested protozoans (Additional file 6: Fig. S4).

### The odds of encountering a parasite in Tingo María versus Andabamba/Marabamba houses

Odds ratio analysis identifies and quantifies associations between the groups tested. Comparisons between houses in Tingo María and Andabamba/Marabamba revealed a significant difference for all helminths (Fisher’s exact test, *P* = 0.0037, OR = 10.5, 95% CI 1.791–52.49) and protozoans (Fisher’s exact test, *P* = 0.0233, OR ≥ 100, 95% CI 1.487–upper bound not estimable) (Table 2). Significant differences were found for helminths for *Ascaris* (Fisher’s exact test, *P* = 0.0037, OR = 16.5, 95% CI 1.8–195.7) and *Trichuris* (Fisher’s exact test, *P* = 0.0421, OR = 8.4, 95% CI 1.0–106.6), which were more prevalent in Tingo María. Among protozoans, a positive association was observed only for *Blastocystis* (Fisher’s exact test, *P* = 0.0421, OR = 8.6, 95% CI 1.2–62.6), which was more prevalent in Andabamba/Marabamba. Due to the small sample size, city parks and playgrounds of Huánuco were not included in this comparison.

**Table 2:** The odds ratio for finding a parasite in Tingo María versus Andabamba/Marabamba houses

Parasite	Tingo María No. of houses	Andabamba Marabamba No. of houses	Odds ratio	*P*-value
Helminths	12*	8	10.50 (1.791–52.59)	*0.0037
*Ancylostoma* species	2	4	0.75 (0.1281–3.859)	0.7598
*Ascaris* *lumbricoides*	6*	1	16.50 (1.829–195.7)	*0.0037
*Necator* *americanus*	0	2	N/A	N/A
*Strongyloides* *stercoralis*	7	0	N/A	N/A
*Taenia* *solium*	0	0	N/A	N/A
*Toxocara* *canis*	1	1	1.615 (0.07940–31.97)	0.7401
*Toxocara* *cati*	0	0	N/A	N/A
*Trichuris* *trichiura*	4*	1	8.4 (1.046–106.6)	*0.0421
All Protozoa	11	22*	> 100 (1.487–upper bound not estimable)	*0.0233
Protozoa not *Acanthamoeba*	11	21	0.1746 (0.01295–1.354)	0.1161
*Acanthamoeba* species	7	17	0.2941 (0.06318–1.184)	0.0906
*Blastocystis* species	10	21*	8.4 (1.046–106.6)	*0.0421
*Giardia * *intestinalis*	3	5	N/A	N/A
Total parasites	14	22	N/A	N/A
Total parasites not *Acanthamoeba*	13	22	0 (0.00–5.727)	0.2036

* Indicates a significant difference (*P* < 0.05)

### Parasites in specific locations of outside built environments

Soil from around the entries, patios, and latrines was collected outside each built environment/house in both Tingo María and Andabamba/Marabamba (Additional file 2: Fig. S2). The odds of detecting any parasite were 3.3 times higher in the patios of Tingo María houses than the entries (Firth’s bias-reduced logistic regression, *P* = 0.0397, OR = 3.297, 95% CI 1.058–10.28). For any helminth, there was 5.9 times higher contamination in the patios than in entries (Firth’s bias-reduced logistic regression, *P* = 0.0065, OR = 5.85, CI 1.64–20.86), with the patios having *A. lumbricoides* 6.7 times higher (Firth’s bias-reduced logistic regression, *P* = 0.047, OR = 6.710, CI 1.03–43.84) than the entries (Table 3). In Andabamba/Marabamba, *Acanthamoeba* species increased 8.64 times in the patios versus the entries for houses (Firth’s bias-reduced logistic regression, *P* = 0.0327, OR = 8.648 (1.194–62.639).

**Table 3:** The odds ratio for finding a parasite in specific locations outside built environments in Tingo María versus Andabamba/Marabamba houses

Parasite	Entry	Latrine	Patio	Odds ratio		*P*-value
Tingo María						
Any parasites	13	11	20	Latrine vs. entry	1.41 (0.446–4.464)	0.5587
				Patio vs. entry	3.297 (1.058–10.276)	*0.0397
				Latrine vs. patio	0.428 (0.125–1.466)	0.1766
Any parasite not including *Acanthamoeba*	10	8	16	Latrine vs. entry	1.2 (0.368–3.914)	0.7625
				Patio vs. entry	2.618 (0.881–7.783)	0.0834
				Latrine vs. patio	0.458 (0.141–1.49)	0.1946
Helminths	4	7	14	Latrine vs. entry	3.025 (0.767–11.932)	0.1139
				Patio vs. entry	5.849 (1.64–20.858)	*0.0065
				Latrine vs. patio	0.517 (0.158–1.693)	0.2758
Protozoa	11	11	14	Latrine vs. entry	1.889 (0.591–6.04)	0.2836
				Patio vs. entry	1.664 (0.571–4.853)	0.3508
				Latrine vs. patio	1.135 (0.356–3.621)	0.8307
Protozoa not including *Acanthamoeba*	8	7	10	Latrine vs. entry	1.346 (0.393–4.612)	0.6362
				Patio vs. entry	1.471 (0.474–4.563)	0.5045
				Latrine vs. patio	0.915 (0.274–3.057)	0.8857
*Acanthamoeba* species	4	6	4	Latrine vs. entry	2.571 (0.617–10.708)	0.1944
				Patio vs. entry	1.043 (0.233–4.673)	0.9556
*Ancylostoma* species	0	1	1	N/A		
*Ascaris lumbricoides*	1	4	7	Latrine vs. entry	5.002 (0.687–36.395)	0.1119
				Patio vs. entry	6.710 (1.027–43.844)	*0.0469
				Latrine vs. patio	0.745 (0.190–2.922)	0.6734
*Blastocystis* species	6	6	10	Latrine vs. entry	1.552 (0.423–5.688)	0.5074
				Patio vs. entry	2.077 (0.637–6.769)	0.2253
				Latrine vs. patio	0.747 (0.219–2.549)	0.6414
*Cryptosporidium* species	0	0	0	N/A		
*Entamoeba histolytica*	0	0	0	N/A		
*Giardia* *intestinalis*	2	2	0	Latrine vs. entry	5.002 (0.687–36.395)	0.1119
				Patio vs. entry	0.193 (0.008–4.449)	0.3039
				Latrine vs. patio	0.745 (0.190–2.922)	0.6734
*Necator* *americanus*	0	0	0	N/A		
*Strongyloides* *stercoralis*	1	4	5	Latrine vs. entry	2.891 (0.529–15.793)	0.2203
				Patio vs. entry	2.591 (0.510–13.170)	0.251
				Latrine vs. patio	1.116 (0.267–4.658)	0.8806
*Taenia* *solium*	0	0	0	N/A		
*Toxocara* *canis*	0	0	1	N/A		
*Trichuris trichiura*	1	1	2	Latrine vs. entry	1.410 (0.130–15.329)	0.7777
				Patio vs. entry	1.797 (0.213–15.160)	0.5899
				Latrine vs. patio	0.785 (0.091–6.772)	0.8254
Andabamba/Marabamba						
Any parasites	11	8	13	Latrine vs. entry	0.95 (0.17–5.305)	0.9536
				Patio vs. entry	1.174 (0.244–5.656)	0.8415
				Latrine vs. Patio	0.809 (0.148–4.438)	0.8076
Any parasite not including *Acanthamoeba*	11	8	9	Latrine vs. entry	0.95 (0.17–5.306)	0.9538
				Patio vs. entry	0.437 (0.1–1.91)	0.2714
				Latrine vs. patio	2.174 (0.434–10.891)	0.3451
Helminths	2	0	1	Latrine vs. entry	0.235 (0.009–6.173)	0.3849
				Patio vs. entry	0.491 (0.054–4.491)	0.5287
				Latrine vs. patio	0.478 (0.016–14.637)	0.6725
Protozoa	11	8	13	Latrine vs. entry	0.95 (0.17–5.305)	0.9536
				Patio vs. entry	1.174 (0.244–5.656)	0.8415
				Latrine vs. patio	0.809 (0.148–4.438)	0.8076
Protozoa not including *Acanthamoeba*	10	8	9	Latrine vs. entry	1.346 (0.393–4.612)	0.6362
				Patio vs, entry	1.471 (0.474–4.563)	0.5045
				Latrine vs. patio	2.173 (0.434–10.89)	0.3451
*Acanthamoeba* species	1	4	8	Latrine vs. entry	5.799 (0.695–48.424)	0.1045
				Patio vs. entry	8.648 (1.194–62.639)	*0.0327
				Latrine vs. patio	0.671 (0.143–3.154)	0.6129
*Ancylostoma* species	0	0	1	N/A		
*Ascaris lumbricoides*	1	0	0	N/A		
*Blastocystis* species	10	8	9	Latrine vs. entry	1.272 (0.237–6.833)	0.7789
				Patio vs. entry	0.585 (0.140–2.443)	0.4626
				Latrine vs. patio	2.173 (0.434–10.890)	0.3451
*Cryptosporidium* species	0	0	0	N/A		
*Entamoeba histolytica*	0	0	0	N/A		
*Giardia* *intestinalis*	1	1	1	Latrine vs. entry	1.381 (0.114–16.679)	0.7996
				Patio vs. entry	0.879 (0.076–10.121)	0.9175
				Latrine vs. patio	1.571 (0.132–18.767)	0.721
*Necator* *americanus*	0	0	0	N/A		
*Strongyloides* *stercoralis*	0	0	0	N/A		
*Taenia* *solium*	0	0	0	N/A		
*Toxocara* *canis*	1	0	0	N/A		
*Trichuris* *trichiura*	1	0	0	N/A		

* Indicates a significant result (*P* < 0.05)

**Fig. 2:**
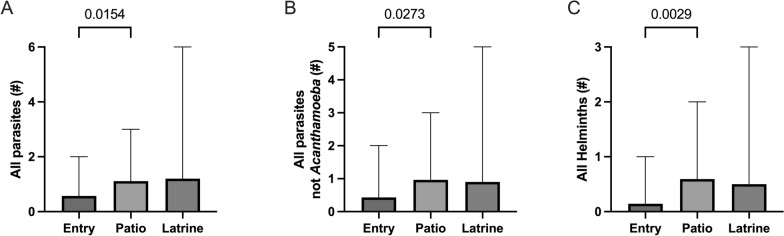
In Tingo María: **A** The total number of different parasite species per outside built environment samples was significantly higher in the patios versus the entries (mean 0.571 vs. 1.11, *P* = 0.0154). **B** Not including *Acanthamoeba*, all parasite species were higher in the patios than the entries (mean 0.429 vs. 0.963, *P* = 0.0273). **C** Helminths were the leading group of parasites in the patios versus the entries (mean 0.143 vs. 0.592, *P* = 0.0029) and differed significantly across entries, patios, and latrines (mean 0.50, *P* = 0.0128)

Using pairwise comparison, the Tingo María patios had significantly more parasite species than the entries (Mann–Whitney *U*-test, *U*(55) = 243.5, *Z* = −2.26, *P* = 0.0154). The patios also had significantly more species of all parasites, not including *Acanthamoeba* (Mann–Whitney *U*-test, *U*(55) = 259, *Z* = −2.0, *P* = 0.0273). Similar findings were seen in all helminth species (Mann–Whitney *U*-test, *U*(55) = 232, *Z* = −2.46, *P* = 0.0029) (Fig. 2). Comparing all three sites (entry, patio, latrine) using ANOVA, there was a significant increase in the patio for all helminth species (Kruskal–Wallis *H*-test, *H* = 8.715, d*f* = *2*, *P* = 0.0128, Dunn’s correction entry vs. patio, *P* = 0.0102). No significant parasite differences existed for specific locations in Andabamba/Marabamba (Table 3). There were no significant differences in the DNA concentrations of each parasite near the entries, patios, or latrines.

## Discussion

The findings of this study reveal significant differences in soil parasite prevalence across different environmental and climatic conditions within the Peruvian regions of Tingo María, Andabamba/Marabamba, and Huánuco. This spatial variation in parasitic contamination underscores the role of environmental factors, such as climate and soil type, in shaping the distribution and density of STH and protozoan pathogens. Such environmental specificity has implications for parasitic risk assessments, especially as global climate change could further influence these distributions [13]. Many of these parasite species are zoonotic and could contribute to soil contamination of outdoor built environmental locations, limiting parasite control efforts [13].

The odds ratio analysis revealed that Tingo María, characterized by a humid rainforest climate, showed higher contamination rates for helminths, particularly *A. lumbricoides* and *T. trichiura*, compared to the drier Andabamba/Marabamba region (Table 2). This outcome aligns with previous literature, suggesting that moist and warm conditions are more conducive to the survival and transmission of STH, as they favor the development and longevity of parasite eggs and larvae [14, 15]. In contrast, protozoan species, specifically *Blastocystis*, were more prevalent in the arid Andabamba/Marabamba region (Table 2), suggesting that certain protozoans may thrive in less humid environments or persist in humid microclimates [16, 17]. Parasite prevalence and burden differed significantly within specific outdoor locations (patio, entry, and latrine) around homes, with the odds of encountering parasites being notably higher in patios than entries, especially helminths in the more humid Tingo María. Conversely, protozoa in the drier Andabamba/Marabamba region suggest that arid conditions may create different microenvironments that selectively favor certain protozoans [18, 19]. This spatial distribution suggests that patios are a high-traffic area in each outdoor built environment, which can support the persistence and infectivity of helminth eggs and larvae. These findings emphasize the importance of spatial considerations in parasitic risk assessments, as different outdoor areas around homes can have varying levels of contamination; this has important implications for targeted anti-parasitic intervention strategies in high-risk zones.

The study’s use of qPCR methodology contributed to the sensitivity and specificity of parasite detection, highlighting the presence of low parasite burdens that might be undetectable by traditional microscopy in stool samples [4, 20]. This increased sensitivity of qPCR is particularly valuable for assessing low-level environmental contamination, allowing for a more comprehensive understanding of parasitic presence and distribution across different microenvironments within households and parks [12, 21]. This study also emphasizes the significance of cumulative parasite burden in environmental reservoirs for public health by identifying multiple parasites within single samples and documenting polyparasitism [5].

While the high prevalence of parasitic DNA in soil samples from Tingo María and Andabamba/Marabamba suggests potential health risks for residents, especially in settings with inadequate sanitation, the findings also indicate that public health interventions must be tailored to the specific environmental and socioeconomic characteristics of each region [12, 22]. For instance, interventions in tropical, humid areas may need to prioritize STH control measures, whereas protozoan monitoring may be more critical in drier, highland environments. Furthermore, longitudinal studies could assess how ongoing climatic shifts—such as increasing temperatures, altered rainfall patterns, and extreme weather events—affect the dynamics of soil parasite communities over time [23].

This study is limited by its relatively small sample size. Although it provides valuable initial insights, it may not fully capture the breadth of environmental variation in parasitic prevalence across Peru. In addition, variability in the DNA extraction process may result in inaccurate prevalence or abundance estimates despite internal controls. Further confounders include the lack of socioeconomic data between the study sites, which can impact parasite contamination [12]. As the qPCR was initially developed for stool studies, we realize that using it for soil studies may provide limitations. While the primer/probe DNA combinations attempted the highest specificity, many animal and non-pathogenic-to-human parasites have similar DNA sequences and may give a false positive result. Future studies with larger sample sizes and additional sites across varied ecosystems and different seasons will be needed to build a more comprehensive parasitic risk map.

## Conclusions

These findings highlight the utility of qPCR as a diagnostic tool for environmental surveillance of parasites, offering a robust approach for monitoring contamination levels and assessing potential public health risks in diverse ecological contexts. This study supports the need for continued monitoring and adaptive public health strategies that consider environmental niches and changing climatic conditions to effectively mitigate soil-transmitted parasite transmission.

## Supplementary Information

Additional file 1.

Additional file 2.

Additional file 3.

Additional file 4.

Additional file 5.

Additional file 6.

## Data Availability

No datasets were generated or analyzed during the current study.
