# Supplementary material for: Parasite contamination of soil in different Peruvian locations and outside built environments
Source: Parasit Vectors. 2025 Apr 5;18:134. doi: 10.1186/s13071-025-06762-7 (PMC11972504; doi:10.1186/s13071-025-06762-7)
Supplement: Supplementary file 2 — Additional file 2. [file 13071_2025_6762_MOESM2_ESM.docx]

**Supplemental information**


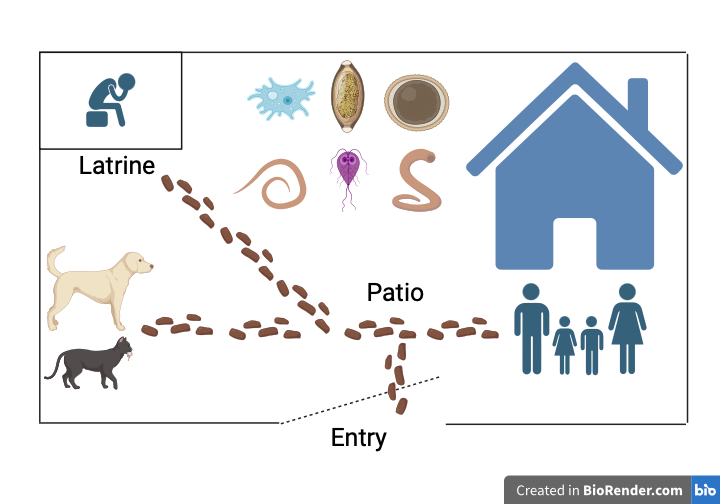
Additional File 2: Fig. S2. Outdoor built environment layout with latrine, patio, and entry soil contamination sites.
