# Supplementary material for: Parasite contamination of soil in different Peruvian locations and outside built environments
Source: Parasit Vectors. 2025 Apr 5;18:134. doi: 10.1186/s13071-025-06762-7 (PMC11972504; doi:10.1186/s13071-025-06762-7)
Supplement: Supplementary file 3 — Additional file 3. [file 13071_2025_6762_MOESM3_ESM.docx]

**Supplemental information**

Additional File 3: Table S1. Environmental Microbiology Minimum Information (EMMI) for qPCR.

| **Environmental Sampling** | **qPCR** |
| --- | --- |
| Up to 50 grams | Target genes (supplemental table 2) |
| Peru  Tingo María  Andabamba/Marabamba  Huánuco | Hold stage 95°C, 20 sec; Amplification Denaturation 95°C, 1 sec; Annealing 60°C, 20 sec. |
| August, 2023 | 2x TaqMan® Fast Advanced Master Mix (Applied Biosystems, Foster City, CA) 3.5 µl |
| Stored at 4°C and DNA extracted within 1 month | 2 µl of template |
| Exogenous DNA was used as an internal control to validate the extraction method. All samples had the internal control detect via qPCR | Primers were used at 900 nM (Thermofisher)  Probe was used at 100 nM (Thermofisher) |
| **Sample Treatment** | QS7 Pro Fast Real-time PCR System (Applied Biosystems, Waltham, Massachusetts, USA) |
| Soil washed with PBS and 0.05% Tween 20 | 2 µl of PCR-water was used as a negative control |
| Flotation with 35.6% Sodium Nitrate solution | Plasmids containing target parasite gene sequences was used as positive control |
| **Sample Reduction** | An exogenous DNA internal control was tested and all samples tested positive for the internal control Ct median  33.80 (31.94 - 37.08). |
| Samples are concentrated by a factor of 500 | **Analysis - qPCR** |
| **Nucleic Acid Extraction** | Positive control standard curves were performed in duplicate |
| MP fastDNA Spin kits for soil | Samples were tested in single |
| DNA eluent is 100 µl and stored at -20°C | All positive controls were compared to a set of known Ct values and were all within 5% range |
|  | Lowest standard measured was approximately 10 fg |
|  | Automatic baseline and a threshold of 0.40 was used for all parasites |
