# Supplementary material for: Parasite contamination of soil in different Peruvian locations and outside built environments
Source: Parasit Vectors. 2025 Apr 5;18:134. doi: 10.1186/s13071-025-06762-7 (PMC11972504; doi:10.1186/s13071-025-06762-7)
Supplement: Supplementary file 4 — Additional file 4. [file 13071_2025_6762_MOESM4_ESM.docx]

**Supplemental information**

Additional File 4: Table S2. Target regions, primer sequences, and probe sequences by parasites for DNA amplification.

| Parasite | Target region | Forward primer sequence (5’ to 3’)  Reverse primer sequence (5’ to 3’)  Probe sequence (5’FAM to 3’) |
| --- | --- | --- |
| *Acanthamoeba* species | 18S rRNA | CCCAGATCGTTTACCGTGAA TAAATATTAATGCCCCCAACTATCC  CTGCCACCGAATACATTAGCATGG |
| *Ancylostoma duodenale* | ITS-2 | GAATGACAGCAAACTCGTTGTTG ATACTAGCCACTGCCGAAACGT ATCGTTTACCGACTTTAG |
| *Ascaris lumbricoides* | ITS-1 | TGCACATAAGTACTATTTGCGCGTAT  CCGCCGACTGCTATTACATCA  GAGCCACATAGTAAATT |
| *Cryptosporidium* species | DNA-J like protein | AACTTCACGTGTGTTTGCCAAT  CCAATCACAGAATCATCAGAATCG  CATATGAAGTTATAGGGATACCAG |
| *Blastocystis* species*.* | 16s rRNA | AGTAGTCATACGCTCGTCTCAAA  TCTTCGTTACCCGTTACTGC  CGTGTAAATCTTACCATTTAGAGGA |
| *Entamoeba histolytica* | 18S rRNA | GTTTGTATTAGTACAAAATGGCCAATTC  TCGTGGCATCCTAACTCACTTAGA  CAATGAATTGAGAAATGACA |
| *Giardia intestinalis* | 16S rRNA | CATGCATGCCCGCTCA  AGCGGTGTCCGGCTAGC  AGGACAACGGTTGCAC |
| *Necator americanus* | ITS-2 | CTGTTTGTCGAACGGTACTTGC  ATAACAGCGTGCACATGTTGC  CTGTACTACGCATTGTATAC |
| *Strongyloides stercoralis* | 18s rRNA | GAATTCCAAGTAAACGTAAGTCATTAGC  TGCCTCTGGATATTGCTCAGTTC  ACACACCGGCCGTCGCTGC |
| *Toxocara canis* | ITS-2 | GCGCCAATTTATGGAATGTGAT  GAGCAAACGACAGCSATTTCTT  CCATTACCACACCAGCATAGCTCACCGA |
| *Toxocara cati* | ITS-2 | ACGCGTACGTATGGAATGTGCT  GAGCAAACGACAGCSATTTCTT  TCTTTCGCAACGTGCATTCGGTGA |
| *Trichuris trichiura* | ITS-1 | TCCGAACGGCGGATCA  CTCGAGTGTCACGTCGTCCTT  TTGGCTCGTAGGTCGTT |
| *Taenia solium* | HSR | AGTCAGTCAGTCAGTCAGTCA  CTGTCAAGCTGTCAGGCTGT  TGTCAGTGAGGCGTGTGACAAGAC |
| *Internal Amplification Control* (IAC) | Synthetic sequence | CTAACCTTCGTGATGAGCAATCG  GATCAGCTACGTGAGGTCCTAC  TCGATGCACTCCAGTCCTCCT |
| *ITS = internal transcribed spacer; rRNA = ribosomal RNA  *HSR = High Sequence Repeats | |  |
