# Supplementary material for: Parasite contamination of soil in different Peruvian locations and outside built environments
Source: Parasit Vectors. 2025 Apr 5;18:134. doi: 10.1186/s13071-025-06762-7 (PMC11972504; doi:10.1186/s13071-025-06762-7)
Supplement: Supplementary file 5 — Additional file 5. [file 13071_2025_6762_MOESM5_ESM.docx]

**Supplemental information**

Additional File 5: Fig. S3. Contaminated houses and parks by distinct parasites.
