# Supplementary material for: Parasite contamination of soil in different Peruvian locations and outside built environments
Source: Parasit Vectors. 2025 Apr 5;18:134. doi: 10.1186/s13071-025-06762-7 (PMC11972504; doi:10.1186/s13071-025-06762-7)
Supplement: Supplementary file 6 — Additional file 6. [file 13071_2025_6762_MOESM6_ESM.docx]

**Supplemental information**

Additional File 6: Fig. S4. Parasite DNA burden of all locations.
